# Glioblastoma and new-onset criminal behaviors in a geriatric patient: a forensic-psychiatric case report from Switzerland

**DOI:** 10.3389/fpsyt.2025.1553508

**Published:** 2025-02-26

**Authors:** Alexander J. Smith, Urs Hagen, Barbara Brela, Anna Buadze, Michael Liebrenz

**Affiliations:** ^1^ Department of Forensic Psychiatry, University of Bern, Bern, Switzerland; ^2^ University Institute for Diagnostic and Interventional Neuroradiology, University Hospital Bern, Bern, Switzerland; ^3^ Department of Psychiatry, Psychotherapy and Psychosomatics, Psychiatric Hospital, University of Zurich, Zurich, Switzerland

**Keywords:** new-onset offending, geriatric patient, glioblastoma, forensic psychiatry, stalking

## Abstract

Organic brain disorders (OBD), including rapid-growth cancerous tumors, can have significant neuropsychiatric effects and in some circumstances have led to the manifestation of deviant behaviors that conflict with societal norms. This report describes the case of a geriatric male patient in Switzerland with no prior history of delinquency who in later life repeatedly committed stalking offences and aggressive acts. An initial forensic-psychiatric evaluation diagnosed this individual with persistent delusional disorder based on pronounced symptoms and rigid personality traits; during this assessment, the patient refused neuroimaging scans but later consented to these examinations. Thereafter, these revealed an isocitrate dehydrogenase wild-type glioblastoma and provided critical insights into his behavioral changes. Specifically, the tumor’s location in regions of the brain responsible for executive functioning, emotional regulation, and social cognition likely contributed to the development of delusions and psychosis-like symptoms that ultimately resulted in new-onset delinquency. Thus, this case highlights the multifaceted challenges of OBDs in forensic-psychiatric contexts, accentuating a need for greater awareness and sensitivity towards these conditions, particularly when externalized deviant behaviors emerge in elderly groups that diverge from established patterns.

## 1 Introduction

Organic brain disorders (OBDs) are characterized by structural or functional abnormalities in the brain that affect cognition, individual behaviors, and emotional regulation (1). As neuropsychiatric conditions, OBDs can include traumatic brain injuries (TBIs), neuroinflammatory disorders, and brain tumors, amongst other issues (1). In forensic-psychiatric frameworks, the causal relationship between OBDs and criminality is complex; nevertheless, OBDs might induce neurocognitive deficits and impaired impulse control, which can contribute to conflicts with societal norms and deviant behaviors (2–7). Notably, varying but significant rates of OBDs have been identified in forensic patient samples but they may well remain underdiagnosed in such settings (3).

Whilst TBIs and neuroinflammatory disorders can exhibit fluctuating trajectories and possible remediable outcomes via appropriate interventions, cancerous brain tumors are more likely to entail progressive courses and poorer prognoses (8). Specifically, across the spectrum of OBDs, gliomas (i.e., a type of primary brain tumor arising from glial cells) represent a discrete subgroup, entailing distinctive adverse behavioral and cognitive effects (4, 9, 10). Malignant high-grade tumors like isocitrate dehydrogenase (IDH) wildtype glioblastoma can have aggressive growth patterns and deleterious neurological consequences (9, 10).

Contingent on their site, severity, and infiltration, these tumors can disrupt executive functioning and emotional processing (9). For example, estimates indicate that 80-90% of glioblastoma patients encounter significant impairments in occupational settings and difficulties in day-to-day functioning (10). Equally, previous work has linked glioblastomas to mood issues, personality changes, disinhibition, and the emergence of delusions and other psychosis-like symptoms (4, 9).

Despite this, instances of delinquency where a brain tumor is present have historically been somewhat neglected in forensic-psychiatric literature and wider contexts of practice (6). Accordingly, the complexity of these conditions and their potential connections to criminal acts warrants greater exploration and attention within this subdiscipline and beyond.

## 2 Case description

This case report concerns a geriatric male patient in Switzerland who had no prior offending history but in later life continually committed stalking and aggressive acts. The patient was subsequently diagnosed with an IDH wildtype glioblastoma.

### 2.1 Developmental and psychosocial history

The patient had a stable upbringing, with no early developmental or psychosocial issues and no known parental history of mental illness. Later, following a divorce, the patient faced significant interpersonal challenges. The patient had a history of consistent employment in different jobs, though in his fifties he transitioned into the teaching sector because of persistent backpain but had conflicts with students and colleagues. This contributed to the onset of what was deemed to be a “burnout” and depressive symptoms, resulting in a psychiatric assessment in 2014 where it was concluded that he was unfit to work. During this, the patient underwent a neuroimaging scan that showed no brain pathologies.

Due to these circumstances, the patient encountered monetary difficulties and received assistance from social services. Furthermore, he was described by his relatives as preoccupied with grievances about his legal and financial struggles but he still maintained interpersonal relationships and engaged in social activities; for example, in 2015, he played an instrument in a theatre play and attended dances several times a week.

Equally, according to reports from his family members, he did not exhibit criminal behaviors until around 2020, after which he appeared markedly more erratic. Specifically, the patient’s family noted that he experienced a “change in character” at this time, becoming increasingly confused, distracted, and aggressive, and had begun sending abnormal emails and correspondence.

### 2.2 Offending history

**Figure 1:**
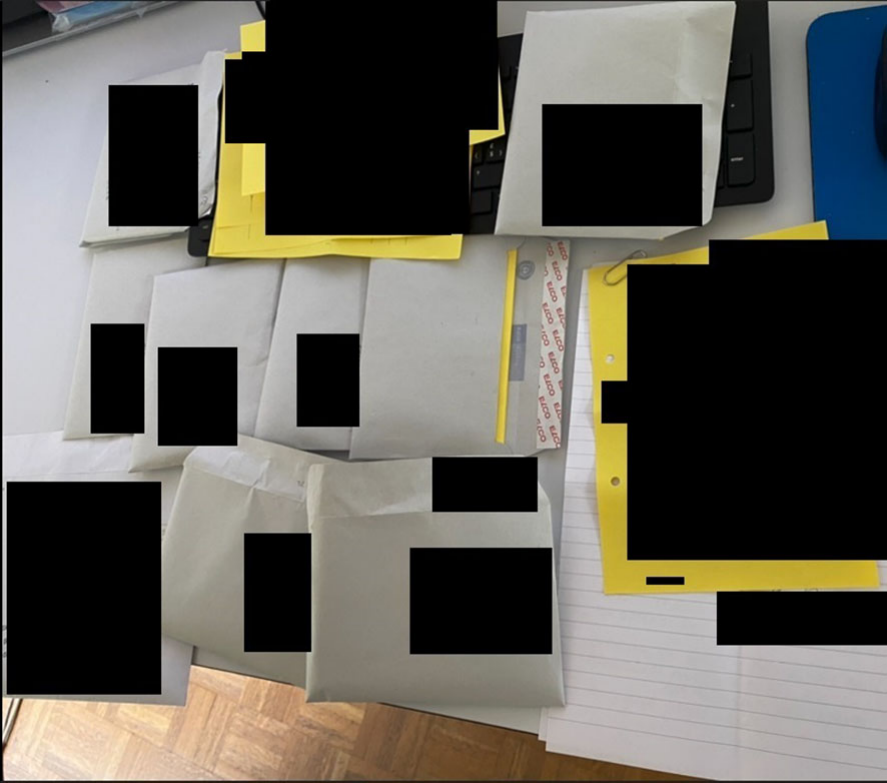
Examples of letters written by the patient received on a single day by the office of the public prosecutor.

In 2021 the patient began exhibiting persistent stalking, harassment, and aggression, despite having no prior history of delinquency. He initially targeted the psychiatrist who had assessed his fitness to work, sending threatening and defamatory correspondence, which he later extended to the psychiatrist’s lawyer. After an official complaint was made to the public prosecutor, the patient was issued with a summary penalty order for defamation in 2022. Thereafter, the patient’s deviant behaviors escalated, with frequent (often daily) letters, emails, and phone calls to family members, public figures, and the authorities (**Figure 1**).

This correspondence was characterized by paranoid, sexualized, and hostile themes and regular accusations of corruption and persecution, growing to increasingly encompass implied threats. Furthermore, between 2022 and 2023, the patient also committed several acts of physical violence.

During this time, he was deemed to be at-risk and was placed under the protection of the local Child and Adult Protection Authority (*Kindes- und Erwachsenenschutzbehörde* - KESB), which assigned him a court-appointed guardian, whom he subsequently began harassing and defaming; the patient referred to this individual as a “*murderous Nazi*”, a “*Nazi expert pig*”, and a “*human torturer*”, alongside other insults. In one email, the patient declared that the guardian would “*only meet me dead for negotiations*”.

At the request of the KESB, the patient underwent psychiatric in-patient treatment for a period of six weeks in 2023. Later, having recurrently threatened his landlady, the patient was evicted from his rental accommodation before his final arrest in September 2023.

### 2.3 Forensic-psychiatric findings

In June 2023, a criminal investigation was initiated and a forensic-psychiatric evaluation was ordered. One of the authors (UH) conducted this evaluation, though the patient refused to undergo any additional neuropsychological exams using standardized assessment instruments or to participate in neuroimaging scans (including both native and contrast-enhanced computed tomography and magnetic resonance imaging).

Based on interviews and collateral information, the patient was provisionally diagnosed with a psychiatric disorder with pronounced delusional symptoms (ICD-10 F22) and a differential diagnosis of a psychotic disorder due to a known physiological condition (ICD-10 F06.2). Additionally, longstanding narcissistic and rigid personality traits were identified.

**Figure 2:**
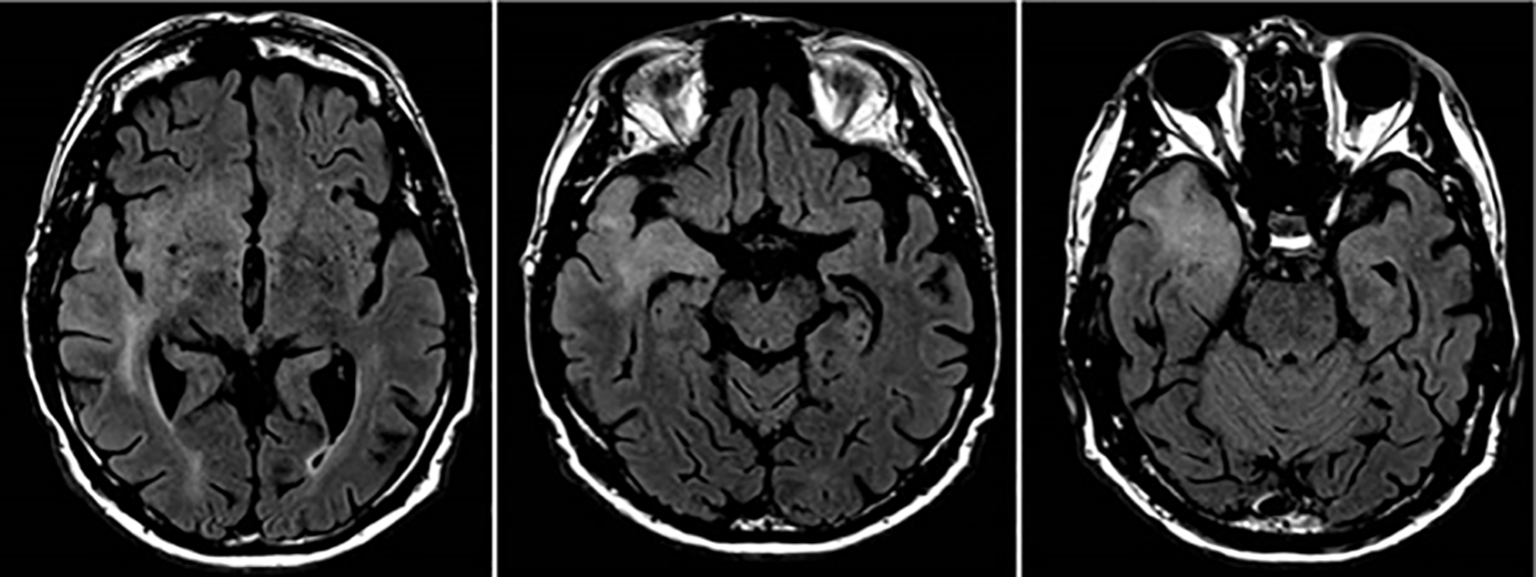
Neuroimaging scans of the patient’s brain tumor showing its infiltration of the cortex and subcortical structures.

Following this, the patient was initially detained in prison before being transferred to inpatient psychiatric care. By early 2024, after experiencing states of tension and sleep problems, he consented to taking antipsychotic medication (haloperidol) and subsequently agreed to undergo neuroimaging, which was carried out thereafter. This revealed diffuse T2/FLAIR hyperintense signal alterations in the right temporomesial, insular, and frontobasal regions of the brain (**Figure 2**).

A biopsy confirmed glioblastoma IDH wild-type WHO grade IV, for which no curative options were available. These results provided critical insights into the escalation of his deviant behaviors, leading to a revised diagnosis of a psychotic disorder due to a known physiological condition (ICD-10 F06.2).

### 2.4 Therapeutic trajectory, prognosis, and current status

Currently, the patient is in a specialized neuropsychiatric ward mandated by pre-trial detention measures receiving palliative care for his glioblastoma, having undergone radiotherapy and chemotherapy. Based on the forensic-psychiatric evidence that substantiated his impaired capacity to stand trial and the severity of his brain tumor, criminal proceedings have been suspended. However, the prognosis remains poor.

Since the diagnosis of his glioblastoma and his subsequent treatment, the patient has exhibited calm and adapted behaviors in the neuropsychiatric ward. Notably, he has sent external correspondence on multiple occasions, which has been polite in tone and devoid of any threatening or sexualized content. He has also expressed his desire to reconcile and spend time with family members.

## 3 Discussion

Although based on a single patient, this case report contributes to the evidence-base around acquired deviant behaviors and conflicts with societal norms that can emerge within the course of glioblastomas. The absence of prior delinquency, the timing of the patient’s behavioral changes, familial accounts, and his trajectory post-diagnosis and after glioblastoma treatment would appear to substantiate a temporal association with the onset of this brain tumor and his criminal acts. Indeed, the manifestation of these offences may well have been the first externalizing sign of this condition, akin to other neuropsychiatric diseases like frontotemporal dementia (11). Specifically, this patient’s trajectory aligns with prior work illustrating substantial neuropsychiatric symptoms from the rapid growth of brain tumors and their infiltration and impact upon key brain regions (4, 5, 9, 12).

As complex phenomena, stalking and harassment can be shaped by multifactorial psychiatric and non-psychiatric risk factors (13–15). Notably, research suggests that older age and an absence of historical violent or sexual crimes predict stalking recurrence and persistence respectively (14). For this individual, the site of the tumor likely disrupted critical neural networks responsible for social cognition (the temporomesial region), self-awareness and emotional salience (the insular cortex), and impulse control, decision-making and inhibitory regulation (the frontobasal areas) (4, 5, 9). Concomitantly, the pathological involvement of these regions could also have precipitated the development of delusions and psychosis-like symptoms that are often present in stalking and harassment incidents connected to psychopathology (13–15).

In this regard, researchers have highlighted how spatially diverse brain lesions can be functionally connected to a network implicated in moral decision-making, value-based reasoning, and theory of mind, which, when disrupted, may contribute to deviant acts (16). Elsewhere, neuroimaging investigations into acquired pedophilia have reported comparable findings, suggesting a causal influence from lesions in the posterior midline structures, right inferior temporal gyrus, and bilateral orbitofrontal cortex (17); profiling studies about acquired pedophilia also identified advanced age and a lack of prior offences as recurrent characteristics of these deviant behaviors (18).

Correspondingly, it is plausible that the patient’s OBD may have amplified his preexisting personality traits into acute and legally problematic behaviors. Whilst the relationships between glioblastomas and personality changes are difficult to delineate, rigidity and narcissism have been linked to stalking offences and were identified in the forensic-psychiatric evaluations in this case (19). Additionally, beyond neurobiological determinants, the patient’s isolation and deteriorating health likely weakened protective social and psychological factors, which might otherwise have mitigated the progression of his stalking and violence (14). Likewise, stressors stemming from financial insecurity and the loss of his employment prior to the criminal acts could have contributed to these externalizing anti-social behaviors; such issues have been identified as risk factors for geriatric delinquency, particularly in lieu of adequate psychosocial support or therapeutic interventions (20).

Nevertheless, this case demonstrates the importance of considering underlying brain abnormalities for new offending in later life, as researchers have previously emphasized for different conditions and crimes (e.g (17–19, 21)). Yet, the reluctance of this individual to undergo neuroimaging due to his symptoms reinforces the difficulties of non-compliance within forensic-psychiatric examinations. Albeit in a single case, adhering to ethical guidelines and respecting patient autonomy can hinder timely and valid examinations, especially amongst elderly populations, potentially informing broader rates of underdiagnosis of OBDs in forensic-psychiatric settings (2, 3).

Accordingly, additional emphasis should be given to OBDs and neural mechanisms in forensic-psychiatric training schemes. Correspondingly, in real-life situations, specialists could prioritize trust-building strategies and conduct continuous follow-up assessments to improve diagnostic accuracy and patient cooperation. Moreover, per recommendations proposed elsewhere, multiprofessional collaborations could help enhance forensic-psychiatric examinations and interventions through holistic knowledge exchanges across allied domains (e.g., neurology, neuroscience, and psychology), particularly in complex clinical cases (21, 22).

As translatable principles, these practices may also yield benefits outside of forensic psychiatry. For general psychiatrists, atypical changes in mood, cognition, or social interactions could be indicators of underlying neurological conditions (6, 12). Consequently, early identification of these issues in general psychiatric services could improve patient outcomes and prevent the possible emergence of more severe or socially harmful acts, as this individual exhibited, which could again be strengthened by multidisciplinary initiatives (22).

## 4 Conclusion

This case report exemplifies the compound intersections between brain tumors and new-onset deviant behaviors that contravene social conventions, particularly regarding the significant behavioral and cognitive changes that can arise in individuals with glioblastoma. Early detection and tailored interventions could augment individual outcomes and lessen delinquency risks and should be underpinned by multiprofessionals collaborations between psychiatrists, neurologists, psychologists, and other disciplines.

Yet, as discussed above, patient non-cooperation can present distinct diagnostic barriers in forensic-psychiatric cases; these could potentially be ameliorated through trust-building and proactive monitoring, especially amongst geriatric groups. In sum, to ensure comprehensive approaches to patient care, this case accentuates a need for increased recognition and understanding towards the neuropsychiatric implications of OBDs in forensic-psychiatric contexts and other related domains.

## Data Availability

The original contributions presented in the study are included in the article/supplementary material. Further inquiries can be directed to the corresponding authors.
